# Evaluation of Anticancer and Antioxidant Activity of a Commercially Available CO_2_ Supercritical Extract of Old Man’s Beard (*Usnea barbata*)

**DOI:** 10.1371/journal.pone.0146342

**Published:** 2016-01-08

**Authors:** Ana Zugic, Ivica Jeremic, Aleksandra Isakovic, Ivana Arsic, Snezana Savic, Vanja Tadic

**Affiliations:** 1 Institute for Medicinal Plant Research “Dr. Josif Pancic”, Belgrade, Serbia; 2 Institute of Rheumatology, University of Belgrade, Belgrade, Serbia; 3 Institute of Biochemistry, School of Medicine, University of Belgrade, Belgrade, Serbia; 4 Department of Pharmacy, Faculty of Medicine, University of Nis, Nis, Serbia; 5 Department of Pharmaceutical Technology and Cosmetology, Faculty of Pharmacy, University of Belgrade, Belgrade, Serbia; Cairo University Faculty of Pharmacy, EGYPT

## Abstract

There is a worldwide ongoing investigation for novel natural constituents with cytotoxic and antioxidant properties. The aim of this study was to investigate chemical profile and stated biological activities of the supercritical CO_2_ extract (SCE) of old man’s beard compared to the extracts obtained using the conventional techniques (Soxhlet extracts and macerate). The most abundant compound identified was usnic acid, which content was inversely proportional to the polarity of the solvent used and was the highest in the SCE, which was the sample revealing the highest cytotoxic activity in tested tumor cell lines (B16 mouse melanoma and C6 rat glioma), with lower IC_50_ values compared to pure usnic acid. Further investigations suggested both SCE and usnic acid to induce apoptosis and/or autophagy in B16 and C6, indicating higher cytotoxicity of SCE to be related to the higher degree of ROS production. A good correlation of usnic acid content in the extracts and their antioxidant capacity was established, extricating SCE as the most active one. Presented results support further investigations of SCE of old man’s beard as a prospective therapeutic agent with potential relevance in the treatment of cancer and/or in oxidative stress-mediated conditions.

## Introduction

Lichens are symbiotic organisms consisting of a fungus and one or more photosynthetic partners. Over the past decades, they have been intensively investigated mainly due to the production of the characteristic and unique secondary metabolites, almost exclusively found in these symbiotic organisms, which have been related to a variety of their natural roles, as well as beneficial biological activities, based on which they have had a long history of utilization as folk remedies [1,2]. Accordingly, in recent years efforts have been made in testing and developing biomaterials containing lichen-isolated natural compounds for human use [2].

Old man’s beard or *Usnea barbata* (L.) Weber ex F. H. Wigg. (Parmeliaceae) belongs to the genus Usnea traditionally used in Asia, Africa and Europe for pain relief and fever control [3]. This lichen has allegedly been used by Hippocrates to treat urinary complaints and in folk medicine of South Africa for the treatment of wounds, while its recommended usage is in the treatment of mild inflammation of the oral and pharyngeal mucosa, as suggested by the monograph of German Commission E [3–5]. Contemporary investigations revealed that, due to the high concentration of usnic acid, old man’s beard displays a wide variety of biological properties attributed to this dibenzofuran derivative [6]. Therefore, in a modern-day development of pharmaceutical and cosmetic industry, extracts of old man’s beard are used primarily as a source of usnic acid [3].

On the other hand, it is well-known that the extraction procedure used for the isolation of substances of natural origin is important in terms of extract purity and the yield of targeted compound(s), which is further connected to the biological activities of the extract [7]. Therefore, in recent years extraction using supercritical fluids, such as CO_2_ (i.e. supercritical CO_2_ extraction) has received increasing attention, having a number of advantages over conventional solvent extraction methods, primarily due to the possibility of providing extracts of higher purity (i.e. without toxic organic solvent residues), with no degradation of active principles and/or their higher yield [8].

In this context, the aim of the current study was to investigate chemical profile and biological activities of the supercritical CO_2_ extract (SCE) of old man’s beard in an attempt to contribute to its potential usage as anticancer and antioxidative agent. For the purpose of comparison, stated investigations also included the extracts of old man’s beard obtained by the conventionally used solvent extraction techniques (Soxhlet extracts and macerate). Cytotoxic activity of the investigated extracts was evaluated in the following tumor cell lines: B16 mouse melanoma and C6 rat glioma and also the non-tumor cells (HaCaT normal human keratinocytes). Observed cytotoxic effect of the extracts was additionally assessed for apoptosis and autophagy through analysis of number of cells in different phases of cell cycle and formation of acidic cytoplasmic vesicles, respectively. Additionally, the stated extracts were submitted to bioactivity evaluation by measuring their *in vitro* antioxidant potential. Moreover, we found it appropriate to correlate specified biological activities with the content of phenolic compounds and in particular to usnic acid content.

## Materials and Methods

### Chemicals and reagents

Analytical grade reagents ethanol, ether, methanol, Folin–Ciocalteu reagent, sodium bicarbonate, gallic acid, H_3_PO_4_, acetate buffer, 2,4,6-tripyridyl-s-triazine (TPTZ), HCl, FeCl_3_·6H_2_O, FeSO_4_·7H_2_O, Butylated hydroxytoluene (BHT), DPPH, dimethyl-sulfoxide (DMSO), Triton-X were purchased from Sigma Aldrich, Germany. Acetonitrile (HPLC grade) was purchased from Merck, Germany. Water (HPLC grade) was produced from double distilled water using Simplicity^®^ UV Water Purification System (Millipore, France). Reference HPLC standard usnic acid (purity>98%) was purchased from Santa Cruz Biotechnology, USA.

### Preparation of the investigated extracts of old man's beard

Lichen *U*. *barbata* (old man's beard) was collected in the Former Yugoslav Republic of Macedonia (GPS N41 53.5764 E21 33.6348) on 15^th^ of October 2009. The stated location did not require specific permission, since it was not a part of a national park (or other protected area of land), nor the private land. The lichen was identified at the Faculty of Biology, University of Belgrade (voucher of specimen No. 16390/10.04.2010.). In addition, our field studies did not involve endangered or protected species.

Investigated samples were following extracts of old man's beard: SCE (E1), Soxhlet extracts (ether fraction (E2) and ethanol fraction (E3)) and macerate (E4). Despite the fact that E2 and E3 were fractions of the Soxhlet extract, we named them extracts to simplify the presentation of results.

E1 was purchased from Flavex, Germany and according to manufacturers’ claims it was obtained by the method of supercritical CO_2_ extraction [drug: extract ratio 62–100:5]. E2 and E3 were obtained by repeated continuous extraction using Soxhlet apparatus. For E2, 5.0 g of dried *U*. *barbata*, grinded to the size of 180 meshes was measured in each of the five thimbles used, covered with appropriate amount of ether and extracted until ether discoloration. Thereafter, the same procedure as for E2 was utilized with ethanol 96.3% (v/v), using thimbles with plant material already extracted with ether, in order to obtain E3. For the preparation of E4, 93.91 g of dried lichen grinded to the size of 180 meshes was covered with 600 mL of ethanol 70% (v/v), macerated for 24 hours and then filtered. After all extractions, liquids were evaporated using rotary evaporator Buchi R 114, USA, yielding 2.04, 6.71 and 5.41% residues for E2, E3 and E4 respectively, expressed as dry matter.

### Chemical characterization of the investigated extracts of old man's beard

#### Determination of total phenolic content

Total phenolic content was determined using Folin–Ciocalteu reagent, according to the method previously described [9]. Shortly, 100 μL of the methanolic solution of the precisely measured weight of the investigated samples E1, E2, E3 and E4 (0.108, 0.112, 0.284, 4.030, 3.780 and 6.130 mg/mL, respectively) were mixed with 0.75 mL of Folin–Ciocalteu reagent (previously diluted 10-fold with distilled water) and allowed to stand at 22°C for 5 min; 0.75 mL of sodium bicarbonate (60 g/L) solution was added to the mixture. After 90 min at 22°C, absorbance was measured at 725 nm. Gallic acid (0–100 mg/L) was used for calibration of a standard curve. The calibration curve showed the linear regression at r>0.99, and the results were expressed as milligrams of gallic acid equivalents per gram of sample dry weight (mg GAE/g DW).

#### HPLC analysis

HPLC analysis of the investigated extracts was performed using Agilent HPLC model 1200; column Zorbax Eclipse XDB-C18 600 Bar (4.6 x 100 mm, 1.8 μm). The mobile phase A consisted of 99% H_2_O and 1% H_3_PO_4_, while B was acetonitrile. Flow rate was 0.1 mL/min, and elution was as follows: 11–55% B, 0–5 min; 55–80% B, 5–10 min; 80% B, 10–12 min; 80–100% B, 12–20 min; 100% B, 20–35 min, 100–11% B, 35–40 min, 11% B, 40–55 min. Investigated samples (in triplicate) were prepared using the following procedure: 5.0, 7.1, 18.9 and 61.3 mg of E1, E2, E3 and E4, respectively were dissolved in 50.0 mL of methanol, then filtered through 0.45 μm PTFE syringe filters into glass HPLC vials and analyzed as described above. The components were identified by relevant retention times and spectral matching. Once spectra matching succeeded, results were confirmed by spiking with respective standard to achieve a complete identification by means of the so-called peak purity test. Quantification was performed by the external calibration with standards.

### Evaluation of cytotoxic activity of the investigated extracts of old man's beard

#### Cell cultures

Mouse melanoma B16 cell line (Cat. Number 92101203) and rat glioma C6 cell line (Cat. Number 92090409) were purchased from European Collection of Cell Cultures (ECACC) and were maintained at 37°C in a humidified atmosphere with 5% CO_2_. C6 cell were cultivated in Hepes-buffered RPMI 1640 supplemented with 2 mM glutamine, 10 mM sodium pyruvate, and 10% fetal calf serum-FCS, while B16 were cultivated in DMEM supplemented with 2 mM glutamine and 10% FCS. HaCaT cell line (normal human keratinocytes obtained from CLS-Cell Lines Service, Eppelheim, Germany) was generous gift from Prof. Andra Jorg, Division of Biophysics, Research Center Borstel, Leibniz-Center for Medicine and Biosciences, Borstel, Germany. HaCaT cells were cultured in DMEM supplemented with 10% FBS, 4 g/L glucose, L-glutamine (2 mM) and 5000 U/mL penicillin, 5 mg/mL streptomycin solution.

Cells were prepared for experiments using standard trypsinization procedure with trypsin/EDTA and seeded in a 96-well-bottom plates for viability assays and in 6-well-bottom plates for flow cytometric analyses. Cells were rested for 24 hours and then treated with appropriate extracts and usnic acid. Investigated samples were dissolved in DMSO and diluted in culture medium. Final DMSO concentration did not exceed 0.1% and had no influence on cell viability.

#### Determination of cell viability

Cell viability was measured by using acid phosphatase assay exactly as previously described [10]. Shortly, after 24-h treatment with old man’s beard extracts/usnic acid, adherent cells were washed twice with phosphate buffered saline, and 100 μL of reaction mixture (0.1 M acetate buffer pH 5.5, containing para-nitrophenyl phosphate (PNPP) and 0.1% Triton-X) was added to each well. After 90 minutes, the reaction was stopped by adding 50 μL of 0.1 M NaOH. The absorbance of the developed yellow color, which was directly proportional to the cells viability, was measured by an automated microplate reader at 405 nm. The results were presented as percent of the control value (untreated cells), which was arbitrarily set to 100%.

#### Cell cycle analysis

The cell cycle was analyzed by measuring the amount of propidium iodide-labeled DNA in ethanol-fixed cells, exactly as previously described [11]. Briefly, the red (FL2) fluorescence of propidium iodide-stained DNA was analyzed with an FACSCalibur flow cytometer (BD, Heidelberg, Germany), using a peak fluorescence gate to exclude cell aggregates during cell cycle analysis. DNA fragmentation, as a marker of apoptosis, as well as the proportion of cells in different cell cycle phases, was determined during cell cycle analysis by evaluating the cells (%) in different phases of cell cycle using Cell Quest Pro software.

#### Intracellular acidification measurement by acridine orange staining

The appearance of acidic autophagic vesicles was detected by flow cytometry, as previously described [12]. Shortly, after the treatment, cells were trypsinized, washed and incubated for 15 minutes at 37°C with 1 μM acridine orange. Acridine orange stained nuclei are fluorescent green, while autophagic lysosomes are fluorescent orange/red (acidic pH). The increase in red *vs*. green (FL3/FL1) fluorescence ratio, reflecting autophagy, was determined by flow cytometry and Cell Quest Pro software.

#### Measurement of reactive oxygen species-ROS

Measurement of ROS was performed using the method previously described [13]. Namely, redox sensitive dye dihydrorhodamine-DHR123 was added simultaneously with treatment to cell culture media. After incubation, cells were detached in dark, washed with PBS and intensity of emitted green fluorescence was measured by FACSCalibur flow cytometer (BD) and analyzed with Cell Quest Pro software.

### Determination of antioxidative activity of the investigated extracts of old man's beard

#### Radical-scavenging activity

Radical scavenging activity was assessed using DPPH assay according to the procedure described by Blois, with some modifications [14]. Specifically, increasing concentrations of different samples were prepared in methanol and 400 μL of this mixture was transferred into each test tube containing 3.6 mL of 0.1 mM methanol DPPH solution, yielding the maximum final concentrations of 0.0112, 0.0284, 0.3760, 0.2420 and 0.0108 mg/mL for E1, E2, E3, E4 and usnic acid, respectively. The absorbance was recorded at 517 nm after 30 min incubation at room temperature in the dark, against methanol as a blank. The percent inhibition was calculated against the control solution, containing methanol instead of test solution using the following equation: I = [(A_c_-A_s_)/A_c_] × 100, where I was the inhibition percentage, Ac was the absorbance of the negative control (contained 400 μL of methanol instead of the samples), and As was the absorbance of the sample. The inhibition percentage was plotted against concentration of the samples, and IC_50_ values, determined by linear regression analysis, presented the mean of three determinations.

#### FRAP assay

FRAP assay was conducted in accordance with the previously reported method [15]. In short, diluted extract (100 μL) and 3.0 mL of freshly prepared FRAP reagent (25 mL of 300 mM acetate buffer, pH 3.6 plus 2.5 mL of 10 mM TPTZ solution in 40 mM HCl plus 2.5 mL of 20 mM FeCl_3_·6H_2_O) were mixed. The absorbance was recorded at 593 nm against a blank, containing 100 μL of resembling solvent, after 30 min incubation at 37°C. The FRAP value was calculated from the calibration curve of FeSO_4_·7H_2_O standard solutions, covering the concentration range 25–400 μmol/L and expressed as mmol Fe^2+^/g dry matter.

### Statistical analysis

The statistical significance of the observed differences was analyzed by the Mann-Whitney U-test or by T-test, or ANOVA followed by the Student-Newman-Keuls test using Statistical Package for the Social Sciences-SPSS version 16.0. A value p<0.05 was considered significant.

## Results and Discussion

### Evaluation of the chemical profile of the investigated extracts of old man's beard

**Fig 1:**
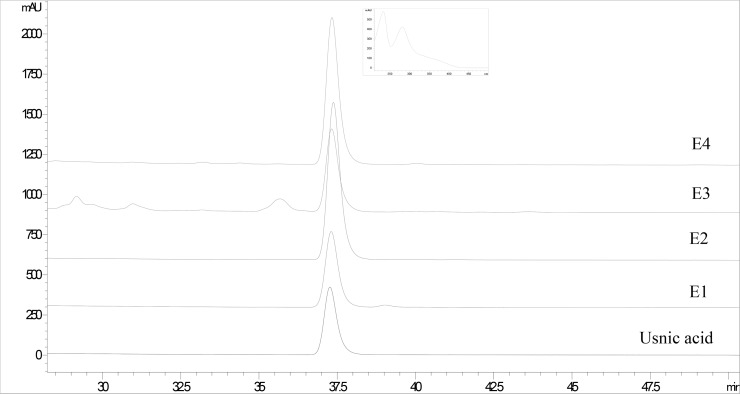
HPLC chromatograms of the investigated extracts of old man’s beard and usnic acid. Supercritical CO_2_ extract (E1); ether fraction of Soxhlet extract (E2); ethanol fraction of Soxhlet extract (E3); macerate (E4).

Since it is known that lichens mainly produce secondary metabolites that are phenolic compounds [16], an initial screening of the chemical profile of the investigated extracts was assessed by total phenolic content determination. As seen in Table 1, total phenolic content ranged from 14.64 mg GAE/g DW in E4 to 164.42 mg GAE/g DW in E1. The lowest content of total phenolic compounds was recorded in the extracts prepared with ethanol, as the most polar solvent used (E3 and E4). Noticeable increase of total phenolic content was observed when the non-polar ether was employed in the extraction process (E2), whiles the highest total phenolic content was detected in the SCE (sample E1) (Table 1). After content of phenolic compounds was established, further detailed chemical analysis was assessed by means of HPLC, which allowed identification of usnic acid as the most abundant phenolic component of the investigated extracts (Fig 1). Moreover, as in the case of total phenolic compounds, usnic acid content (Table 1) was inversely proportional to the polarity of the solvent used in the extraction process [17], extricating SCE (sample E1) as the extract with the highest yield of this dibenzofuran derivative.

**Table 1:** Results of the chemical analysis (total phenolic content (expressed as mg GAE/g DW) and usnic acid content (expressed as % (w/w)) of the investigated old man’s beard extracts (supercritical CO_2_ extract (E1); ether fraction of Soxhlet extract (E2); ethanol fraction of Soxhlet extract (E3); macerate (E4)).

Sample	Total phenolic content (mg GAE/g DW)	Usnic acid content (% (w/w))
**E1**	164.42	81.41
**E2**	144.12	67.09
**E3**	25.70	2.43
**E4**	14.64	1.39

### Evaluation of biological activities of the investigated extracts of old man's beard

#### Evaluation of cytotoxic activity

**Fig 2:**
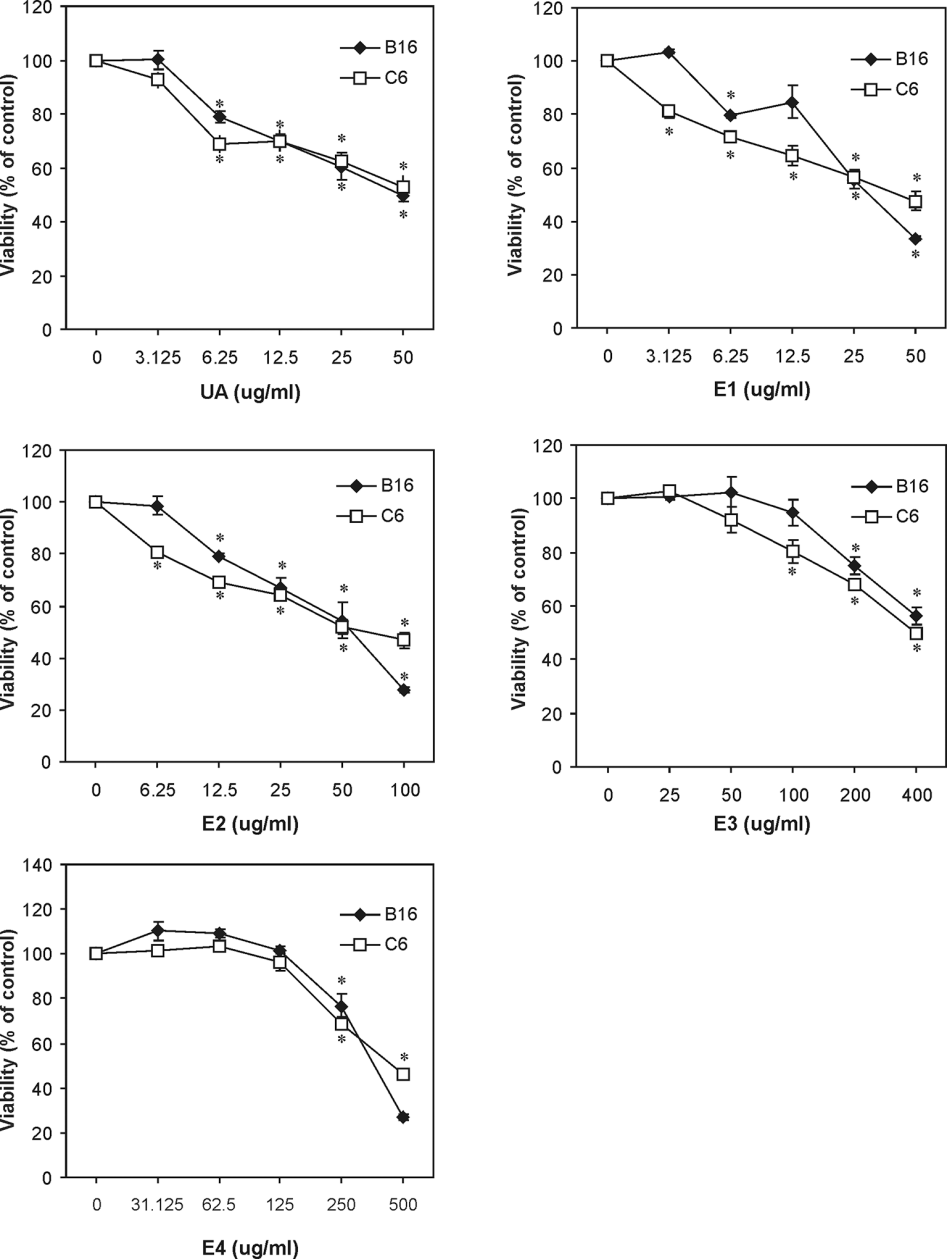
Cytotoxic dose dependent action of old man’s beard extracts/usnic acid. B16 mouse melanoma cells and C6 rat glioma cells were incubated with different concentrations of samples: usnic acid (UA), supercritical CO_2_ extract (E1), ether fraction of Soxhlet extract (E2), ethanol fraction of Soxhlet extract (E3) and macerate (E4). The cell viability was assessed after 24 h by using acid phosphatase test. The results are presented as means ± SD values of triplicate observations from a representative of three independent experiments (*p<0.05 refers to control-untreated cells).

We further aimed at investigating cytotoxic activity of the studied extracts of old man's beard, as well as usnic acid, as the major component identified in the extracts in the first part of the study. Cytotoxic activity was assessed in B16 and C6 cell lines and all the investigated extracts decreased the viability of both stated cells in the dose dependent manner (Fig 2). In general, among the investigated samples, SCE (E1) exerted the highest cytotoxic activity (Table 2), with B16 (IC_50_ 31.21 μg/mL) being more susceptible cell line in comparison to C6 (IC_50_ 43.40 μg/mL). With the exception of E1 having lower IC_50_ values than the ones determined for pure usnic acid, cytotoxic activity of the extracts was in good correlation with their usnic acid content (Tables 1 and 2). Namely, ether fraction of Soxhlet extract (E2) revealed also significant cytotoxic effect to both tested cell lines (Table 2), but this effect was proportionally lower with the lower usnic acid content recorded for this sample (67.09% (w/w)). Following this trend, ethanolic fraction of Soxhlet extract (E3) and macerate (E4), which contained far less usnic acid (2.4 and 1.4% (w/w), respectively) exerted week cytotoxic activity (Table 2). In addition, all tested samples were significantly less toxic to the non-cancer normal human keratinocytes HaCaT cell line, suggesting their selectivity against cancer cells (Table 2).

**Table 2:** IC_50_ values (expressed as μg/mL) in tested cancer (B16 melanoma and C6 glioma) and non-tumor (HaCaT normal human keratinocytes) cell lines treated with the investigated samples: old man’s beard extracts (supercritical CO_2_ extract (E1); ether fraction of Soxhlet extract (E2); ethanol fraction of Soxhlet extract (E3); macerate (E4)) and usnic acid.

Sample	IC_50_ values for tested cell lines (μg/mL)		
	B16	C6	HaCaT
**E1**	31.21	43.40	278.75^a^
**E2**	58.20	69.10	380.31^a^
**E3**	466.10^a^	395.60	543.44^a^
**E4**	391.76	457.24	637.10^a^
**Usnic acid**	49.14	56.83^a^	232.98^a^

^a^ indicating IC_50_ values outside the range of the experimental concentrations

Based on these results, it might be assumed that usnic acid, which was also the major compound identified in all the tested extracts, could be held accountable for the observed cytotoxic activity, additionally taking into account well-established toxic effects of this dibenzofuran derivative in a variety of cancer cells, reported in the literature over the past decades [18–32]. On the other hand, it has been reported that, in some cases, the extracts containing the active substance demonstrated stronger bioactivity than the purified compound alone [33,34]. In our study, SCE of old man’s beard (E1) revealed lower IC_50_ for both tested cell lines (B16 and C6) compared to usnic acid and hence further investigations were performed in order to gain a deeper insight into the mechanisms involved in the demonstrated cytotoxic activity of this sample compared to pure usnic acid.

**Fig 3:**
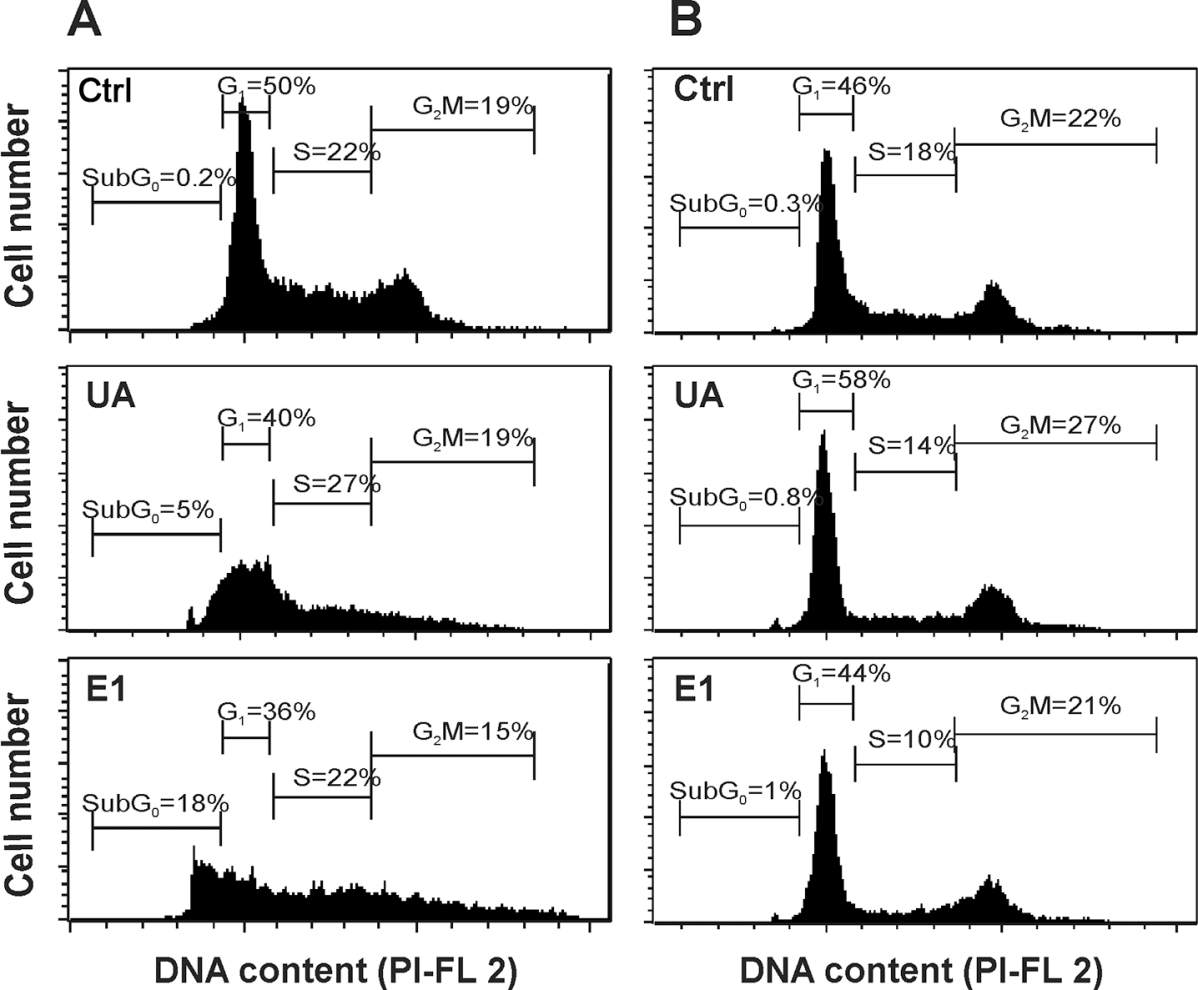
The effects of supercritical CO_2_ extract of old man’s beard (E1) and usnic acid (UA) on the cell cycle progression of B16 (A) and C6 (B) cells. Numbers on histograms represents the percentage of cells in appropriate phase of cell cycle (sub G_0_, G_1_, S and G_2_) under the treatment with UA (25 μg/mL) and the most active extract-E1 (25 μg/mL) for B16 (A) and C6 (B) cells.

Cell cycle analysis revealed significant number of B16 cells treated with both samples (E1 and usnic acid) to show hypodiploid DNA content, indicating DNA fragmentation i.e. apoptosis (Fig 3A). It has been already shown that usnic acid induces apoptosis in HeLa [23], HT-29 and A2780 cell lines [19]. Also, investigation of Rankovic et al. indicated prominent ability of the acetone extract of old man’s beard and pure usnic acid to induce apoptosis in FemX and LS174 cells [18]. In contrast, C6 cells treated with usnic acid and E1 did not show any change in cell cycle analysis (Fig 3B), implying that, in these tumor cells, apoptosis was not the mechanism responsible for the exerted cytotoxic effects.

**Fig 4:**
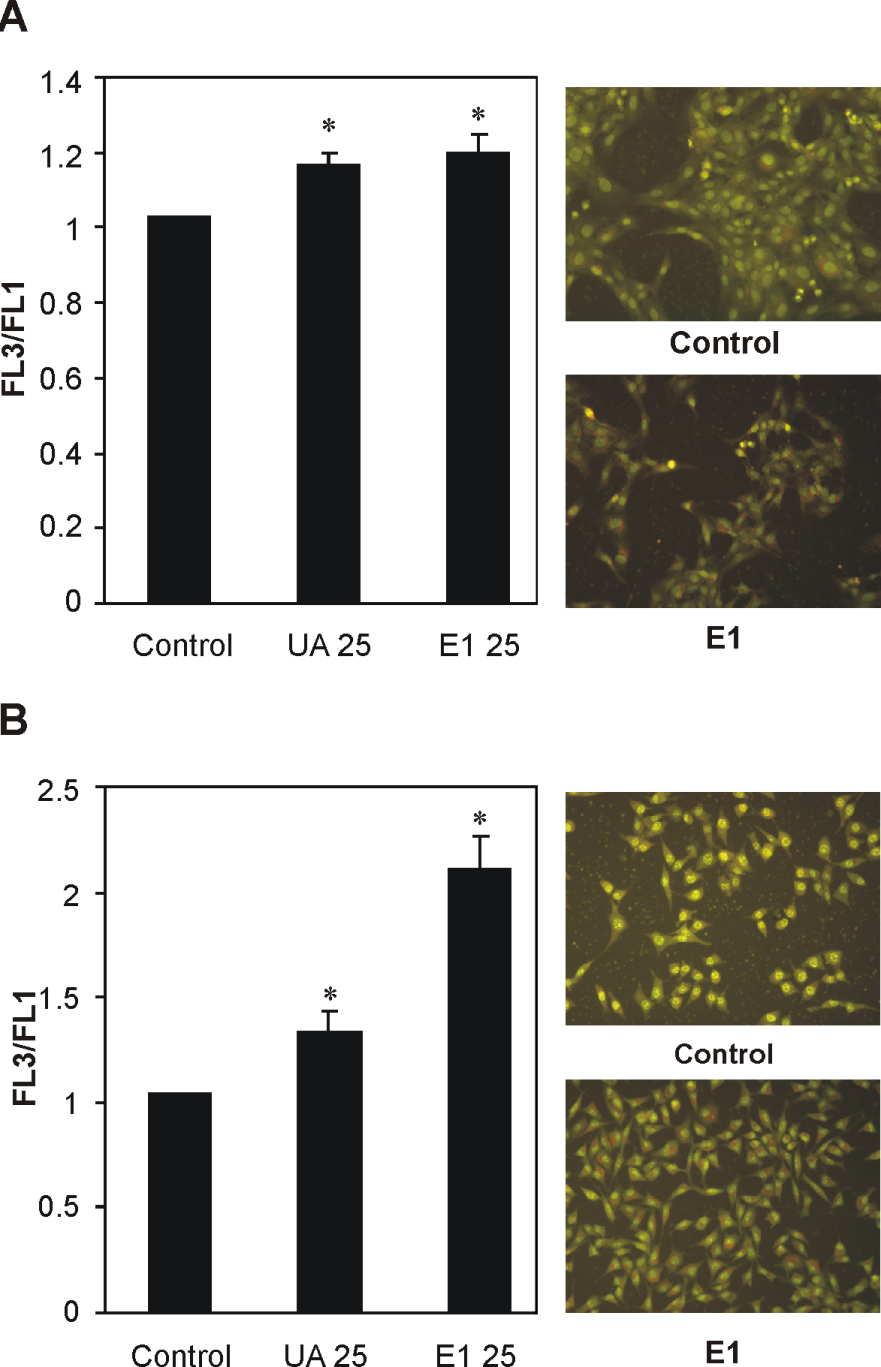
The effects of supercritical CO_2_ extract of old man’s beard (E1) and usnic acid (UA) on the induction of autophagy. On the left, the intracellular acidification was assessed by flow cytometry following acridine orange staining of B16 (A) and C6 (B) cells and is represented by histograms of mean fluorescence FL3/FL1 intensity increase under the 24 h treatment with UA and E1 (25 μg/mL). On the right, the fluorescent micrograph of tumor cells stained with acridine orange indicates the presence of acidic cytoplasmic vesicles in B16 (A) and C6 (B) cells treated with the most active extract-E1. Relative increase in FL3/FL1 fluorescence (left) represents means ± SD values from at least three independent experiments (*p<0.05 refers to control as untreated cells).

However, treatment with both SCE (E1) and usnic acid induced massive formation of acidic cytoplasmic vesicles, a morphological characteristic of autophagy, in treated C6 cells (Fig 4B). Both SCE (E1) and usnic acid also significantly increased acidic cytoplasmic vesicles in B16 cells (Fig 4A). It is well-known that autophagy could serve dual role in antitumor action of cytotoxic drugs: 1) as a pro-survival mechanism of cancers cells, or 2) as an alternative method of cell death *per* se, or tightly coupled to apoptosis/necrosis [35]. With respect to previous inferences in this study, it seems that in the tested tumor cells autophagy had different contributions in the exerted cytotoxicity of SCE of old man’s beard/usnic acid. Namely, in the case of B16 cells, autophagy might have served either as the protector [26] or the promoter of apoptosis [35], whilst in C6 cells it acted as an alternative method of cell death [35]. In this connection, autophagy as a mechanism of cell death was recently described in two cancer cell lines (breast cancer cell lines: T47D and MCF7 and the pancreatic cancer cell line: Capan-2) treated with usnic acid [21].

**Fig 5:**
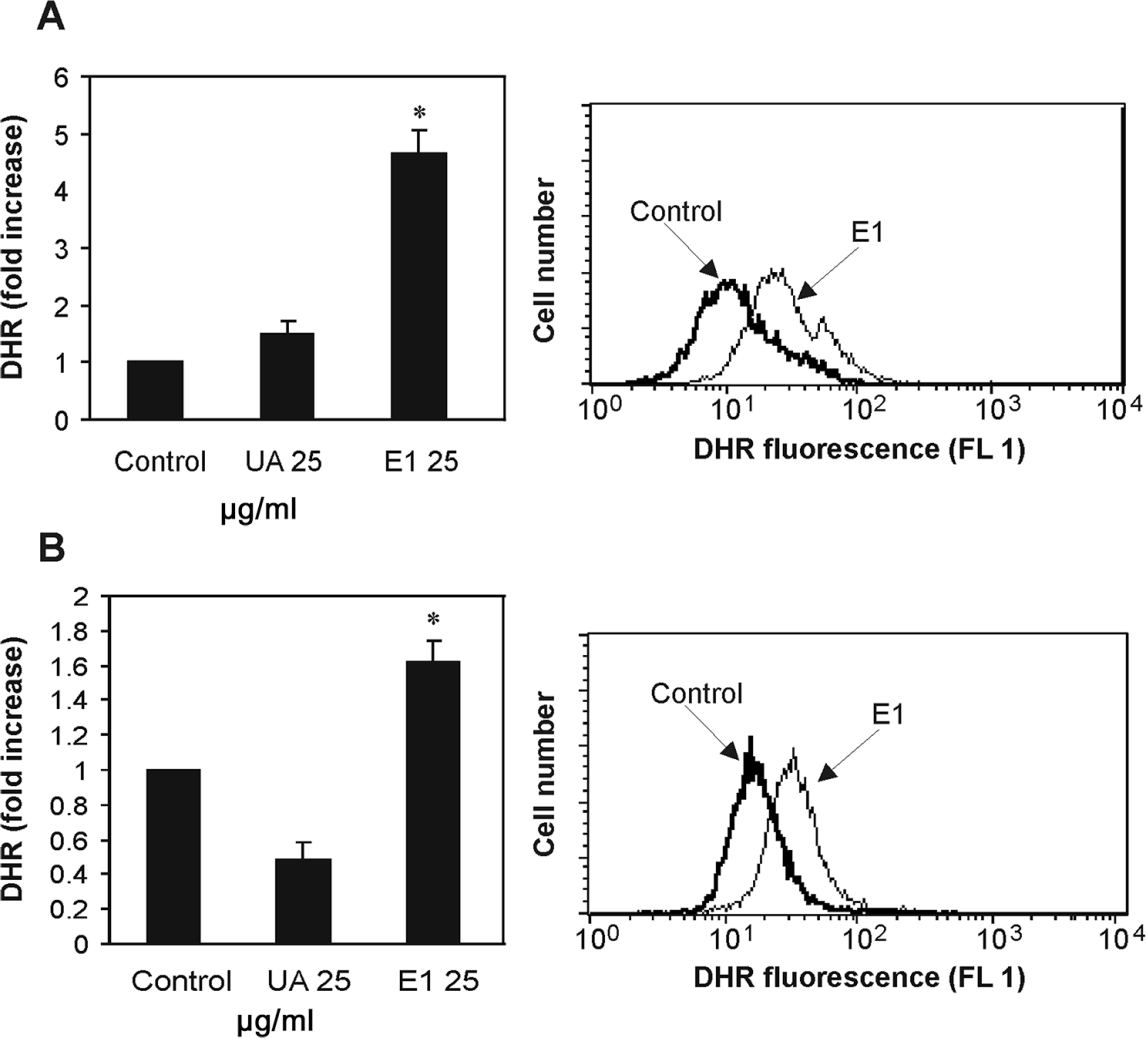
The effects of supercritical CO_2_ extract of old man’s beard (E1) and usnic acid (UA) on the ROS production. B16 cells (A) and C6 cells (B) were incubated with E1 and UA (25 μg/mL) and ROS production was measured after 24 h by flow cytometry. On the left, relative increase in DHR fluorescence (compared to control as untreated cells) under the tumor cells treatment with E1 and UA (concentrations are expressed in μg/mL) is presented as mean ± SD values from three independent experiments (*p<0.05 refers to control-untreated cells). On the right, representative histograms of mean fluorescence intensity increase under the 24 h treatment with most active extract-E1 for both B16 (A) and C6 (B) cells.

Our further experiments revealed significant production of ROS in B16 cells treated with the most toxic extract of old man’s beard (SCE i.e. E1) (Fig 5A). E1 induced significant oxidative stress in C6 cells as well (Fig 5B), but less pronounced than in B16 cells. As opposed to this, usnic acid did not induce neither increased nor decreased production of ROS in tested cells (Fig 5), thus confirming truancy of oxidative stress in the exerted cytotoxicity [23]. It has been well established that oxidative stress may contribute to the cytotoxicity of a substance, throughout several different mechanisms [36]. Therefore, it appears that differences in exerted cytotoxicity of the SCE and usnic acid in both tested cell lines (IC_50_ 31.21 *vs*. 49.14, for B16 and IC_50_ 43.40 *vs*. 56.83 for C6) may be connected to the different degree of ROS production. However, further experiments are needed to support these presumptions and elucidate precise mechanisms involved in ROS induced-cytotoxicity of the tested SCE of old man’s beard.

Overall, SCE of old man’s beard (E1) revealed to be the most toxic substance in both tested cancer cell lines (melanoma B16 and glioma C6), among all the investigated extracts and also pure usnic acid. All tested samples were significantly less toxic to non-cancer immortalized normal skin HaCaT cell line, suggesting their selectivity against cancer cells. Additional examinations suggested the same mechanisms (i.e. apoptosis and/or autophagy) to be involved in the exerted cytotoxicity of both SCE (E1) and usnic acid. In further experiments, however, significant increase of ROS in both tested cell lines was observed after treatment with E1, but not usnic acid. Bearing in mind that oxidative stress may contribute to the cytotoxicity of a substance, observed discrepancy in exerted cytotoxicity of SCE of old man’s beard in comparison to usnic acid in both tested cell lines may be explained, at least, as a function of the different degree of ROS production.

#### Evaluation of antioxidative activity

Many biological effects of the lichen extracts and their secondary metabolites have been related to their antioxidant properties [2]. Hence, further investigations of biological activities of the tested extracts of old man’s beard and usnic acid included evaluation of their antioxidative activity. It has been well established that the antioxidant activity cannot be evaluated using a single test and therefore, two commonly applied assays differing in their working principles, have been employed [37].

One of the methods used for the determination of antioxidant properties of the tested samples was a method of neutralization of DPPH radicals. The principle of this method is that antioxidants react with free radical DPPH (purple colored solution) and by hydrogen donating translate it to a clear DPPH-H, wherein the degree of discoloration indicates the antioxidant potential [38]. In our experiments, none of the investigated extracts, neither usnic acid exhibited a radical scavenging activity, evaluated by means of DPPH assay (data not shown). BHT, used as a reference standard, showed IC_50_ value of 11.2 μg/mL. In contrast to our findings, a recent study reported IC_50_, determined using DPPH assay, for the acetone extract of old man’s beard and pure usnic acid [18]. However, several papers dealing with antioxidant properties of pure usnic acid, imparted this dibenzofuran derivative not to scavenge DPPH radicals, regardless its phenolic nature, suggesting usnic acid not to possess labile hydrogen atoms [2,23,39].

**Fig 6:**
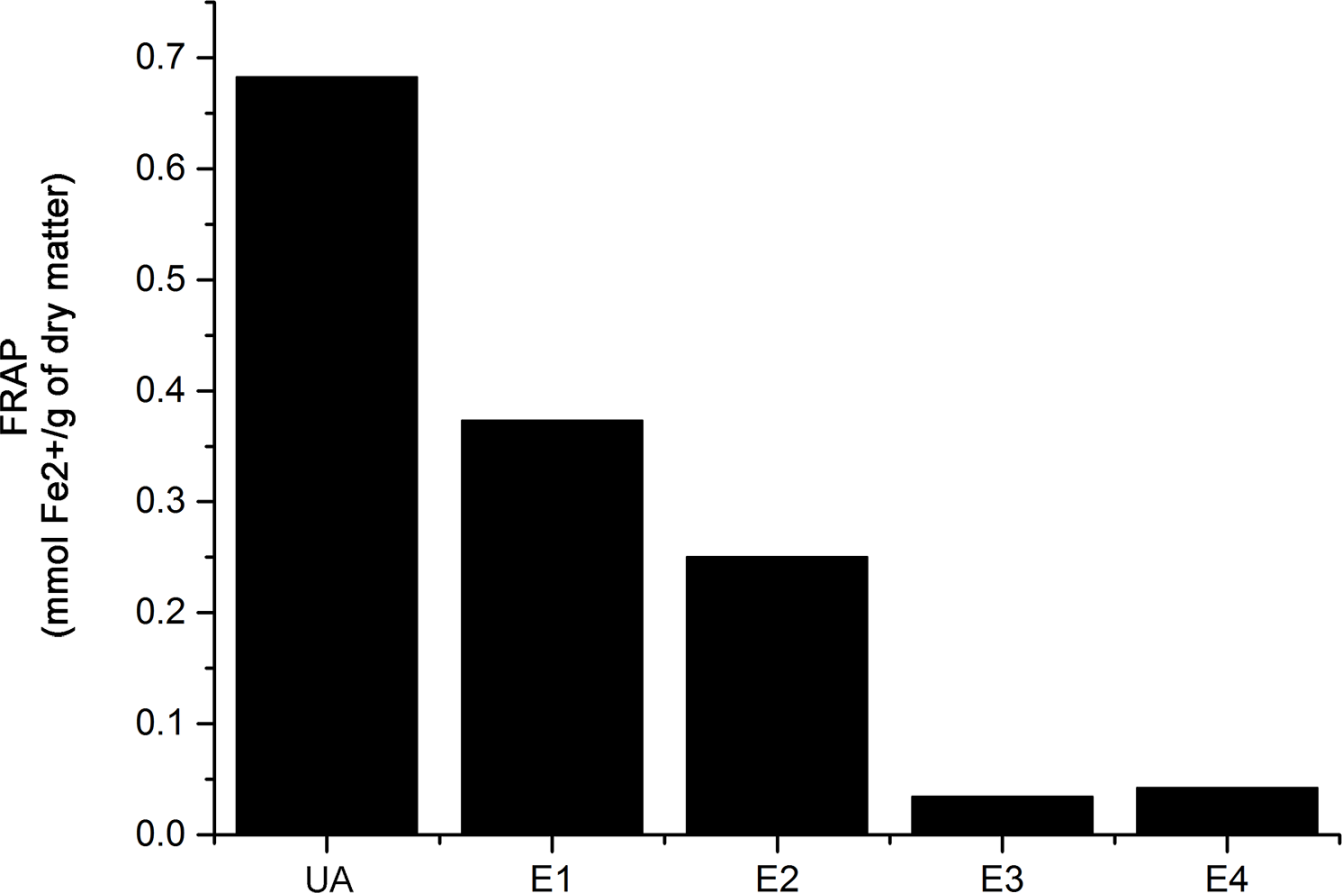
Antioxidant activity of the investigated samples. Usnic acid (UA); supercritical CO_2_ extract (E1); ether fraction of Soxhlet extract (E2); ethanol fraction of Soxhlet extract (E3); macerate (E4) (FRAP values expressed in mmol Fe^2+/^g of dry matter).

The other test used for the evaluation of antioxidative activity was the FRAP assay, which measures the capacity of a substance to reduce the oxidative species by electron transfer, while changing the colour (determined spectrophotometrically), wherein the degree of the colour change is proportional to the substance concentration [38]. Results of the FRAP assay conducted in our study are presented in Fig 6. As seen, a tendency towards grouping of the investigated extracts according to usnic acid content (Table 1) and antioxidant activity assessed by FRAP assay (Fig 6) was clearly noticeable. Namely, SCE (E1) had the best antioxidant potential among the investigated extracts of old man’s beard with FRAP value being almost twice higher in comparison to the ether fraction of Soxhlet extract (E2), followed by drastically weaker antioxidative potential of ethanolic fraction of Soxhlet extract (E3) and macerate (E4) (Fig 6). Such findings are in line with many studies reporting a strong correlation between the content of phenolic compounds and FRAP values [40,41]. With respect to this, FRAP value of pure usnic acid was expectedly higher compared to E1 (Fig 6). Demonstrated antioxidative activity revealed by means of FRAP assay of the tested extracts could be considered moderate compared to BHT, employed as a reference antioxidant, that had a FRAP value of 1.35 mmol Fe^2+/^g. Such findings are consistent with the findings of Rankovic et al., which admittedly used reducing power assay employing ascorbic acid as a positive control [18].

## Conclusion

Usnic acid was the major phenolic compound identified in all the investigated extracts of old man’s beard. Its content was the highest in the supercritical CO_2_ extract (SCE), which further revealed the highest cytotoxic activity in tumor cells B16 and C6. Additional examinations suggested the same cytotoxic mechanisms of both SCE and usnic acid in both tested cell lines, indicating lower IC_50_ of SCE to be related to the higher degree of ROS production. A good correlation of usnic acid content in the extracts and their antioxidant capacity assessed by FRAP assay was established, extricating SCE as the most active sample. Presented results support further investigations of SCE of old man’s beard as a prospective therapeutic agent with potential relevance in the treatment of cancer and/or in oxidative stress-mediated conditions.
